# Beyond global health: Redefining the ‘public’ in public health

**DOI:** 10.1177/14034948221109712

**Published:** 2022-09-16

**Authors:** Mats Målqvist

**Affiliations:** Swedesd – Sustainability Learning and Research Center, Department of Women’s and Children’s Health, University Hospital, Uppsala, Sweden

**Keywords:** Global health, planetary health, public health

## Abstract

The world has seen unprecedented changes over the last 50 years, with enormous gains in human health and living standards. Global public health has been a part of this transition to an interconnected and interdependent world, evolving from a medically based international health perspective to a global health discipline focusing on the social determinants of health and systems thinking. As we now face global challenges such as climate change, the loss of biodiversity and antimicrobial resistance, global public health needs to be transformed yet again. Public health needs to redefine its focus. To expand the scope beyond the anthropocentric – and to include nature and our planet as subjects and not merely resources for human well-being – is of the essence.

The world has seen incredible shifts in public health over the last 50 years. The demographic and epidemiological transitions have changed the patterns of disease and fertility, and living conditions have improved at an unprecedented rate. Human well-being has never been greater and more people than ever before have the opportunity to thrive and develop their potential. An ever-more interconnected world spreads innovations and knowledge at a speed that public health practitioners half a decade ago would never have dreamed of. However, these developments have also created new threats and challenges: persistent inequities and colonial power structures, the destruction of the biosphere and climate change are both real and alarming. The globalised and interlinked world does not only present opportunities, it is also fragile. Small disturbances in one end of the system can develop rapidly and cause major disruptions and, in the worst-case scenarios, even reach catastrophic proportions. Emerging diseases, such as COVID-19, the spread of antimicrobial drug resistance, disruptions in supply chains and the collapse of food systems are only a few of the threats, not only to single individuals, nations or regions, but to all of humanity.

Over the last 50 years, global public health has changed along with these historical shifts. The era of vertical health programmes, focusing one isolated problem at a time, culminated with the fight against smallpox in the 1970s. Even if later being accused of working in ‘silos’, it was an undisputable success when the disease was officially declared eradicated in 1980. This was in a world of ‘us and them’, of rich and poor countries, heavily shaped by the remnants of a colonial world order. International health as a concept, driven by the mission to promote public health in less fortunate settings, created a global health arena characterised by good will and activism. However, it also manifested a world characterised by unequal relations, the disproportionate accumulation of wealth and the maintenance of colonial structures [1]. The perspective was, to a large extent, medical [2], with physicians, nurses and midwifes from the global north travelling to the global south to contribute to faltering health systems and mitigate the lack of resources and capacity.

The Alma Ata Declaration of 1978, a major milestone in the field of public health, tried to set a new direction, emphasising the need to go beyond vertical solutions and instead adopt a more holistic approach. ‘Health for All’ became the slogan and ambitious plans to develop community health care were drawn up. However, realism kicked in as the resources for this project were not there. Furthermore, the neoliberal project to maintain a colonial world order gained traction in the 1980s. This resulted in Structural Adjustment Programs, with the International Monetary Fund and the World Bank putting demands on policy changes in line with a neoliberal agenda as a prerequisite for loans, leaving already weak health care systems in the global south desolate [3]. This, in turn, paved the way for western non-governmental organisations to step in and fill the void as far as possible, manifesting old power relations. The inequalities of the system came into focus and Margaret Whitehead’s seminal writings on health equity added a stronger social dimension [4]. Even if Whitehead’s definition of health equity advocated a shift from vertical thinking to more collaborative efforts, it was still operating within the medical health paradigm, outlining determinants for health as causes of ill health.

In 1986, the Ottawa Charter, the result of the first international conference on health promotion, became a defining moment for global public health. It highlighted health promotion as the key to improving people’s health and shifted the focus from detecting and curing disease to prevention [5]. Furthermore, the Ottawa Charter started a change in discourse that would shift our understanding of health as a concept. The focus on health as something that can be promoted, an asset to be developed, aligned it better to the then 40-year-old World Health Organization definition of health being ‘not merely the absence of disease’, but the presence of well-being [5]. The shift was slow, however, and when the Millennium Development Goals (MDGs) were formulated just before the turn of the century, they still expressed a strong disease-related focus. Even if successful as a policy advocating tool – and MDGs 4, 5, and 6 were the hallmark for international health all through the first decade of the new millennium – the MDGs were heavily criticised [6]. The lack of equity application and the colonial focus on solely the poorer and ‘least developed’ parts of the world exposed how the international health paradigm no longer had a justification [6].

The work of the Commission on the Social Determinants of Health [7] also furthered the thoughts on health equity from Whitehead’s singular focus on the proximal determinants to a widened understanding of the ‘causes of the causes’. When the first conclusions from the Commission on the Social Determinants of Health report were published in 2008, it meant a new era for public health, marking the emergence of global health as a new paradigm, better aligned with the no longer bipolar world of developed and developing countries. Global health also acknowledged the interconnectedness of the world, stressing that ‘we are all in this together’ and that changes in one part of the system will affect other parts of the same. This horizontal approach was visible in the post-2015 agenda, which eventually led up to the Sustainable Development Goals.

The definition of global health has been debated, with its adversaries accusing it of lacking focus and being ‘a theory of everything’ [8]. Critics mean that when ‘health’ becomes defined from a societal perspective, including all and nothing, it becomes impossible to operationalise. However, a common understanding of global health has emerged, defining it as focusing on health equity and transnational health threats [9]. As opposed to its predecessor international health, it acknowledges that inequalities in health outcomes are a general problem and that the health challenges today are not only the concern of populations far away. Global health is not ‘public health somewhere else’ [10]. Widening the definition of health to not only include well-being or the absence of disease, but also to recognise that health is the capacity to develop human potential, opens new avenues for understanding the mechanisms of ill health. Putting the focus on societal power structures as the underlying root causes of health problems has the potential to re-direct attention and priorities.

The world is, however, changing rapidly and global health needs to re-align. Even if most institutions, such as academia and non-governmental organisations, are still struggling to adapt away from the international health perspective, with recent frequent calls and efforts to ‘decolonise global health’, there are calls to further redefine the focus. Efforts such as One-Health, stressing the interconnectedness of, and shared arena between, humans and animals [11], and planetary health, striving to include environmental and biosphere perspectives [12], indicate that the discourse is evolving. Global health, with its understanding of the root causes and influence of underlying structures is still, to a large extent, anthropocentric, with a rights-based approach derived from human rights. Planetary health expands our understanding of this relation beyond humanity [1,13], meaning that humanity is only one entity in a bigger picture, an entity with the power to enhance or destroy the world around itself and, as such, has a special responsibility. Recent advocacy movements point to the need to widen the rights-based approach to also include the biosphere as an entity with rights of its own [14]. This would mean a subjectification of nature and alter our approach to what is the ‘public’ in public health [13].

How public health will evolve in the next 50 years is difficult to anticipate, but given the difficulties of the current challenges we know that it will change. A first step is to conclude the process from international health to global health by embracing the new world order and more actionably address old power structures and world views [1]. It demands the adoption of a truly global approach, stressing the interconnectedness and similarities between contexts, applying research and discourses not only to local, national or regional settings [13]. This also means asking uncomfortable questions about resource and power allocation, which is necessary for the systemic shift needed in years to come [1]. The next steps are to open up the arena and see beyond the anthropocentric view, to acknowledge the impact of humanity as a whole in relation to the planet we are living on and to encourage and incorporate discussions about planetary rights [14]. This new perspective is not only a matter of trustworthiness and authenticity, but a matter of survival, setting the direction for public health for the next 50 years.
